# FuGeF: A Resource Bound Secure Forwarding Protocol for Wireless Sensor Networks

**DOI:** 10.3390/s16060943

**Published:** 2016-06-22

**Authors:** Idris Abubakar Umar, Zurina Mohd Hanapi, A. Sali, Zuriati A. Zulkarnain

**Affiliations:** 1 Department of Wireless and Communication Technology, Faculty of Computer Science and Information Technolog, University Putra Malaysia, Serdang 43400, Malaysia; brossadiq@gmail.com (I.A.U.); zurinamh@upm.edu.my (Z.M.H.); zuriati@upm.edu.my (Z.A.Z.); 2 Research Centre of Excellence for Wireless and Photonic Network (WIPNET), Department of Computer and Communication Systems Engineering, Faculty of Engineering, University Putra Malaysia, Serdang 43400, Malaysia; aduwati@upm.edu.my

**Keywords:** wireless sensor network, node selection, malicious nodes, routing protocol, security, fuzzy logic, geographic forwarding

## Abstract

Resource bound security solutions have facilitated the mitigation of spatio-temporal attacks by altering protocol semantics to provide minimal security while maintaining an acceptable level of performance. The Dynamic Window Secured Implicit Geographic Forwarding (DWSIGF) routing protocol for Wireless Sensor Network (WSN) has been proposed to achieve a minimal selection of malicious nodes by introducing a dynamic collection window period to the protocol’s semantics. However, its selection scheme suffers substantial packet losses due to the utilization of a single distance based parameter for node selection. In this paper, we propose a Fuzzy-based Geographic Forwarding protocol (FuGeF) to minimize packet loss, while maintaining performance. The FuGeF utilizes a new form of dynamism and introduces three selection parameters: remaining energy, connectivity cost, and progressive distance, as well as a Fuzzy Logic System (FLS) for node selection. These introduced mechanisms ensure the appropriate selection of a non-malicious node. Extensive simulation experiments have been conducted to evaluate the performance of the proposed FuGeF protocol as compared to DWSIGF variants. The simulation results show that the proposed FuGeF outperforms the two DWSIGF variants (DWSIGF-P and DWSIGF-R) in terms of packet delivery.

## 1. Introduction

Routing and security are two of the most prominent research challenges in WSN that have gained much ground, due to the emergence of Cyber-Physical Systems (CPS) and Internet of Things (IoT) that employ sensor devices for communication. Although the sensor has resource constraints in computation, memory, bandwidth and energy, a routing protocol is required to provide a safe and reliable path to ensure an end to end communication in a network [1]. This path is built by selecting appropriate nodes that are traversed from one end of the network to another. However, several security approaches used by routing protocols are inefficient in mitigating spatio-temporal attacks. This is because of the high resource consumption of the pertinent mechanisms employed for the security solutions on the sensor node. Therefore, resource bound security solutions are highly needed to provide minimal active security protection.

Resource bound security solution is the process of using improved protocol’s operation semantics to provide adequate security. It can be kept active or activated when needed to optimize protocol performance while minimizing security threats using minimal resource consumption [2]. This solution was proven successful in [2] and has been applied to various routing protocols in [2,3,4] to mitigate spatio-temporal attacks. Spatio-temporal attacks are attacks plotted in a network after extensive correlation analysis of an existing communication pattern in the network. In other words, attackers are able to determine temporally the presence of data in nodes and importantly, to predict the position (spatially) of the next forwarding node [5]. Attackers successful in such siege can further decide to drop all (blackhole), partially drop (grayhole), or Insert data into the packet. Any of the attacks can drastically degrade the performance of a network to an undesirable level.

In [3], a DWSIGF protocol was proposed to mitigate the black hole attack. It introduced a dynamic collection window period to create a possible time shift in the protocol’s semantics. The protocol improved performance in protection against security attacks. However, the utilization of a distance based parameter (only) for forwarding node selection in the network has simplified spatio-temporal predictions, causing strategic placement of malicious nodes to succeed in enabling substantial packet losses in the network. Furthermore, it achieved a poor trade-off between its security performance and base performance, such that either inefficient utilization of the constrained resources is made to achieve the minimal security or the base performance is maintained by efficiently utilizing the resources at the expense of security.

In this paper, we propose a Fuzzy-based Geographic Forwarding protocol (FuGeF) to improve node selection. The protocol first utilizes three parameters: remaining energy, connectivity cost, and progressive distance for node selection, then, employs a Fuzzy Logic System (FLS) for decision-making. The goal of FuGeF is to identify appropriate forwarding nodes that would mitigate packet loss as well as provide a better trade-off between security and base performance. Extensive simulation experiments have been conducted to evaluate the performance of the proposed FuGeF with the DWSIGF protocol. The results obtained show that the FuGeF achieves a higher performance in terms of packet delivery ratio and minimizes the possibility of choosing an attacker as compared to DWSIGF protocol.

The rest of the paper is organized as follows: Section 2 briefly discusses some related works. Section 3 describes the base protocol, while Section 4 describes our proposed protocol. Performance evaluations of the base and proposed protocol were discussed in Section 5 and Section 6 presents the conclusion and future works.

## 2. Related Works

An ideal protocol, despite node’s constraints (energy, processing power, bandwidth, and storage) is expected to coordinate communication activities to achieve high delivery ratio, and low latency using the least energy possible [6]. Thus, to fulfil these requirements, there is the need for a protocol to have an efficient forwarding strategy. This strategy is known to dominate the node selection process that influences network performance. In this section, we review some forwarding or node selection strategies that were employed in several routing protocols.

One of the earliest works on node selection employs the Most Forward within Range (MFR) or greedy forwarding strategy [7] to select next hop nodes. The strategy selects a node that makes the highest advances (distance) towards a destination within its range, during the routing process. It minimizes the number of hops and energy consumption. Due to these advantages, several protocols such as CBF [8], GeRAF [9], BLR [10], SBGR [11], IGF [12] and BOSS [13] have adopted the MFR’s selection strategy. However, this strategy is affected by interference and holes which lead to packet losses due to longer hop distance between nodes or failed hop.

In [14], a Nearest with Forward Progress (NFP) was proposed to mitigate the interference and hole problem. NFP selects immediate neighbors’ nodes to the sending nodes. In other words, it selects nodes within its range which made shorter advances away from the sending node towards a destination. Similarly, Random Progress (RP) forwarding strategy was also proposed in [15] to mitigate interference. RP selects nodes randomly within its range as long as they posses a distance value which shows progress towards the destination. Both strategies increased packet delivery due to shorter hops chosen, which were invulnerable to interference as seen in GPER [16], SIGF [2] and DWSIGF [3] . However, these protocols suffer an increase in latency and energy consumption due to the shorter hops utilized for routing.

In [17], Compass routing was proposed to efficiently select the next hop node considering an angle criterion in addition to the distance criterion. The forwarding strategy selects a next hop node using MFR or NFP, as well as the angle of deviation of each node within the forwarding range from the sending node. The node with minimum deviation angle from the sending node is chosen as the next hop node. Protocols such as HGR [18] and PFR [19] utilized this form of node selection to improve packet delivery and reduce energy consumption. This was because minimum deviation angle provides a slightly direct line of contact to the sending node, since such nodes (that are on a direct line of contact with each other) are less prone to interference and path dilatation. However, the use of MFR and NFP forwarding strategies in its selection leads to interference, increase energy consumption and delay, respectively.

In [20], Energy Efficient Geographic Forwarding Strategy (EEGFS) was proposed to select the next hop node with consideration to Packet Reception Rate (PRR) and distance transversed towards the destination. The EEGFS first computes the product of PRR and distance transversed towards the destination. The protocol then selects the node with maximum product value as the next hop node. The strategy achieved higher delivery rate with a relatively lower value of energy and delay. This was because the highest values used for the node selection were mostly dominated by nodes which achieved optimal forwarding distance. However, this distance as described by Zuniga and Krishnamachari in [21], falls within a translational region where link quality has high variance (including good and weak links), which if not intelligently chosen, may lead to poor network performance.

Similarly [22] proposed a Geographic Routing with Environmental Energy Supply (GREES) to improve energy efficiency and packet delivery. The GREES selection criteria use parameters such as channel link condition, packet progress to a destination, residual energy of node and environmental energy supply for next hop selection. The next hop node was computed based on GREES-L and GREES-M. The GREES-L and the GREES-M utilize the linear and multiplicative functions of the parameters (respectively) in order to balance the geographical advance efficiency per packet transmission and the availability of receiving nodes. The protocol achieved the increase in energy efficiency and packet delivery at the expense of increase in end to end delay. However, the GREES was unable to balance between geographical advance efficiency per packet transmission and the availability of receiving nodes because packets may have to travel along some links of weaker quality or travel longer hops to get to their destinations.

In [23], a Multi-Criteria Decision Analysis (MCDA) based method for optimization was adopted for node selection. Multi-Criteria Decision Routing protocol (MCDR) was proposed to improve on the longevity of the network and traffic distribution. It employs distance and remaining energy as criteria for node selection. The approach achieves node selection by the addition of criterion values after each criterion was multiplied with a fixed value known as a weight. However, the flexibility in weight assignment affects the optimization process, causing output utilized for node selection to skew towards an intended objective due to an assigned weight value. This hinders other performance metrics as they tend to portray a poor trade-off in the metrics.

Some earlier works employed the use of Computational Intelligent Systems (CIS) for node selection. Among the numerous CIS, Fuzzy logic system (FLS) has emerged as one of the most commonly used decision-making algorithms to improve network performance. The FLS, which is based on mathematical proofs, accepts values (parameter) from nodes as inputs, where it computes and infers output values. The computation involves converting input values (crisp input) into a fuzzy set and processing it using a series of IF-THEN rules, which are intended to mimic human reasoning. The processed fuzzy set is converted back into output values (crisp output). In most cases, the node with the highest output value is selected as the next hop node. This fuzzy logic based node selection strategy is utilized in protocols such as SFRRP [24], DSR [25], BCFL [26], FMAR [27], NORIA [28], and [29], to mention a few. The flexibility in the choices of input parameters for the FLS makes it tuneable and adaptive to different application needs, thus, improving the targeted performance metrics. On the other hand, improper selection of these parameters may hinder or cause unbalanced performance between metrics, resulting in poor performance of the network.

Despite attempts to optimize trade-offs in performance metrics of these protocols, only a few of the protocols considered were intended for security purposes. This is because the security mechanisms in protocols demand higher utilization of the constrained sensor resources (which if depleted, may compromise the entire network performance). Resource bound security solutions proposed the alteration of protocol routing semantics to implement security mechanisms which impedes the effect of attacks on a network as well as maintains optimal performance. This improved approach is seen in SBGR [11], SIGF [2] and DWSIGF [3] routing protocols mentioned earlier. The Resource bound security approach is among the first few attempts that were proposed to provide a better trade-off between security and performance of a protocol.

SBGR [11] was proposed to mitigate Sinkhole and Sybil attacks in a network. It employs the MFR forwarding strategy for node selection as well as other improvements in the protocol semantics for its security operation. The modification includes propagating the message beyond the region of attacker influence to abates the Sybil attack and verification of node propagation location to subdue the Sinkhole attack. The verification is done by temporarily storing each node message along with its location and ensuring the attacker does not claim to provide the highest advances towards the destination. These modifications improved the protocol’s security performance but it, however, consumed more resources (memory and energy) and exhibited MFRs drawbacks. Furthermore, strategic placements of malicious nodes may hinder the protocol’s performance as the MFR selection of next hop node may be easily predicted.

SIGF [2] was proposed to mitigate Blackhole and Sybil attacks. It employs both MFR and RP forwarding strategies in SIGF-priority and SIGF-random respectively, for node selection. The protocol’s semantics was modified by introducing a sampling process which lasted for a fixed window of time. The fixed window period allowed the selection of a non-malicious node from gathered response obtained from next hop nodes (forwarding candidates) thus abating the attacks. The protocol (SIGF-priority) achieves efficient resource utilization but it, however, maintains a consistent routine that is highly predictable and vulnerable to malicious nodes placed strategically along its routinely followed path. SIGF-random erratic behavior achieves minimal security at a heightened cost of resource utilization.

DWSIGF [3] builds on SIGF [2]. It improves on the sampling process introduced in SIGF by proposing a dynamic window period to create a possible time shift in the protocol’s semantics, thus, causing a dynamic routine or uncertainty during the selection process. An attacker in this instance is unable to respond timely for sampling within the window period. This improvement enabled DWSIGF to outperform SIGF. However, DWSIGF also employs the MFR and RP forwarding strategy for node selection (DWSIGF-P and DWSIGF-R, respectively). The strategies, despite the improved routing semantics, still suffer from SIGF drawbacks.

In this paper, our approach builds on the DWSIGF protocol. We leverage the use of three parameters which are progressive distance, remaining energy, and connectivity cost, as well as a FLS for node selection. These parameters describe features external to a node, as well as influence distribution of traffic load among the nodes. The FLS is used to evaluate the chance value of each next hop node based on its three criteria values. The values obtained are used to determine the most appropriate forwarding node for the routing process. Additionally, dynamism introduced in DWSIGF was maintained with a slight modification such that a shift in the proposed protocol semantics is also achieved. The main aim of the proposed FuGeF is to achieve efficient security while maintaining an acceptable level of base performance.

## 3. The DWSIGF Protocol

**Figure 1:**
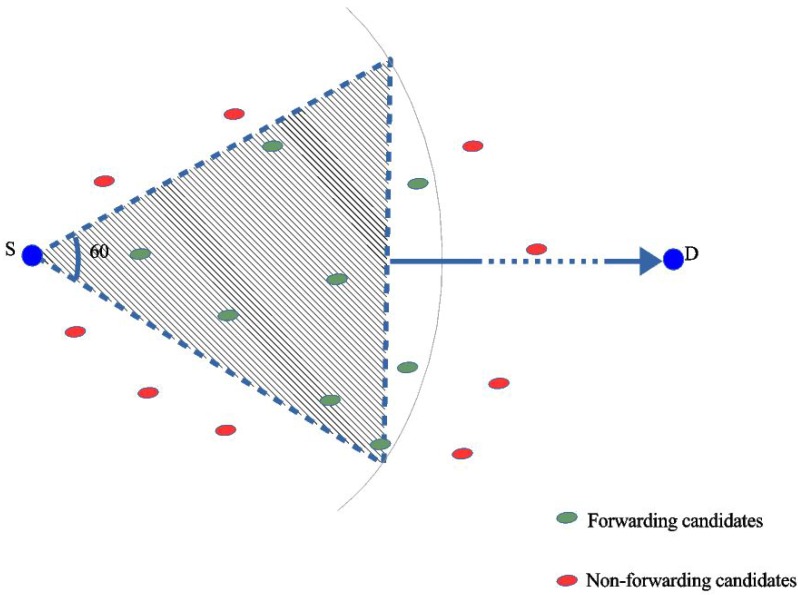
Forwarding area.

This section describes the DWSIGF protocol. The protocol utilizes both MAC and network layer for routing. The MAC (IEEE 802.11DCF) uses a four-way handshake semantics for the node to node coordination within a 60-degree sextant, centered on a direct line with the destination, as shown in Figure 1. The handshake is initiated when the Sender S’s NAV timer is zero and an idle channel is sensed for a DIFS time period, before broadcasting an Open Request to Send (ORTS) signals (containing its location and destination location) to nodes within its broadcast range. The nodes within the broadcast range consist of the forwarding candidates (within the 60-degree sextant area) and non-forwarding candidates shown in Figure 1. On receiving the broadcast, the non-forwarding candidates suppress their responses by setting their NAV timer in accordance with 802.11 semantics to avoid interference. On the other hand, the forwarding candidates respond by setting up a CTS response time. When the candidate’s CTS response time expires, a CTS signal containing candidate’s location is sent to the ORTS sender S as the response [2,3,12,30].

The sender S collects multiple responses within the dynamic collection window period. The window period creates a possible time shift in the protocols semantics [3,4]. The responses gathered within the collection window period are immediately sampled and a node is selected based on either DWSIGF-P or DWSIGF-R forwarding strategy. The strategies use Increasing Distance Towards the Destination (IDTD), which is a parameter that defines a node’s progress towards the destination and maps out its position on the network. DWSIGF-P utilizes the greedy forwarding strategy, which means the node with the highest IDTD is selected as the forwarding node. On the other hand, DWSIGF-R employs a loop-free random forwarding strategy, it randomly selects any node as long as it has a positive IDTD value. On selecting the forwarding node, sender S proceeds in accordance with the 802.11 DCF semantics (ORTS =>CTS=>DATA=> ACK) using an isolated channel with the receiver [3,4].

DWSIGF mitigates selection of attacker to minimize packet losses. However, the utilization of IDTD parameter (only) for forwarding node selection in both strategies(DWSIGF-P and DWSIGF-R) has resulted in the simplification of spatio-temporal predictions. This has allowed malicious nodes to succeed in causing substantial packet loss in the network.

## 4. The Proposed FuGeF Protocol

This section presents the proposed FuGeF routing protocol. It is a modification of the DWSIGF [3] that improves on security as well as ensures efficient utilization of constrained resources (maintains base performance). The FuGeF introduces three parameters comprising remaining energy, connectivity cost, and progressive distance, as well as a Fuzzy Logic System (FLS) to enable appropriate node selection in a network, which is presented in Section 4.1 and Section 4.2, respectively.

**Figure 2:**
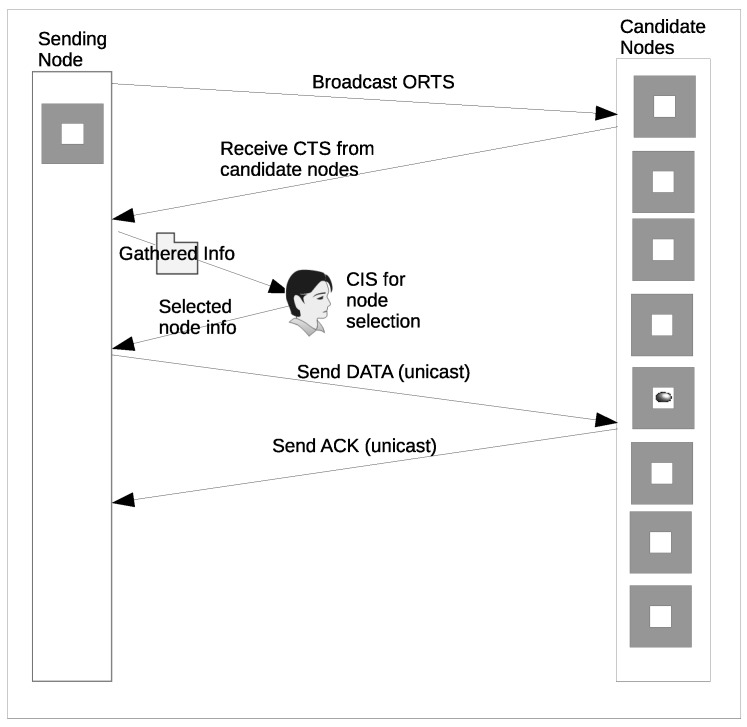
Forwarding process.

In contrast to DWSIGF, the proposed FuGeF uses individual CTS reply received by the ORTS sender S, which contains location information and remaining energy. The sender S employs the location information to compute a node’s progressive distance and connectivity cost. The computed values, together with the remaining energy, are passed as inputs to the FLS for processing as shown in Figure 2. The processing is in line with the FLS semantics explained in Section 4.2. The node with the highest output value (chance value) is regarded as the most appropriate forwarding candidate and is selected as the next hop node. The use of these parameters together with FLS causes spontaneity in both the forwarding node selection and the routing pattern, thus making it difficult to perform spatio-temporal predictions on the network. This lowers the chances of selecting malicious nodes despite their strategic placement.

Additionally, FuGeF employs a pseudorandom based dynamic collection window to maintain the dynamism of DWSIGF. However, FuGeF’s pseudorandom based window ranges between two fixed intervals. These intervals ensure a slight shift in the protocol semantics such that at least a single CTS response is collected for processing. This dynamic limit-based feature, together with FLS consensus, increases unpredictability in node selection during the routing process. The Algorithm for the proposed FuGeF is described in Algorithm 1.

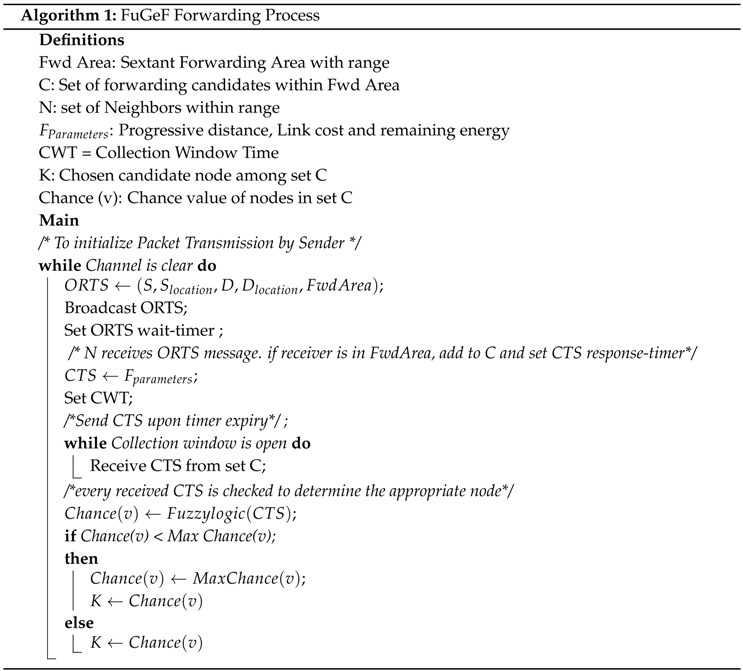


### 4.1. Design Parameters in FuGeF

In this section, we detail more on the parameters used in our proposed protocol.

1. *Progressive distance (Pd)*: This is the Euclidean distance from the sending node to each candidate nodes within the broadcast range. It enables the sender to determine the position of a nodes within its range and their progress toward the destination (Unlike IDTD). This information also allows the sender to determine its link strength relative to the candidate nodes within its range.
2. *Connectivity cost (C)*: Modeled as a function of distance as shown in Equation (1), the connectivity cost denotes the loss in link/connection quality between the sending node and the forwarding candidates, due to the gap existing between the nodes. This gap determines the weak and good connection quality in the network. The larger the gap between the nodes, the weaker is the connection.
  (1) $$C=\frac{1-Pd}{Radius}$$
  The parameter is derived from Equation (2) , which has been adopted from He *et al.* in [30] as follows:
  (2) $$Q=\frac{\left(Wp\times \frac{1-Pd}{Radius}+Wr\times rand\left(\right)\right)}{\left(Wp+Wr\right)}$$
  where Wp is the weight of progress, Wp is the weight of randomness, Pd is the Progressive distance and Radius is the Range. The cost *Q* was also utilized in FuGeF, as it determines the distribution of nodes within the 60-degree sextant region. *Q* depends on both Wp and Wr. The higher the value of Wp, the more it enhances the few nodes with the most progressive distance value (leading to shorter routes to the destination) with the least traffic distribution and lower connectivity cost. On the other hand the higher the value of Wr, the more distributed are the traffic to more nodes, leading to better load balance and higher connectivity cost [30]. Information regarding node’s connectivity cost is useful towards enhancing node selection. In this paper, to obtain candidate nodes that have made significant progress with links that are good enough to prevent excessive packet loss when routing, we assumed Wp = Wr.
3. *Remaining Energy (RE)*: Otherwise known as residual energy is considered as one of the most important parameters in WSN’s technology because it determines the lifetime of a node in a network. Using the first order radio model [31], remaining energy of a node is calculated as
  (3) $$RE=NodesInitalEnergy-(E_{Tx}+E_{Rx})$$
  where $E_{Tx}$ and $E_{Rx}$ are the Transmit and Received Energy, respectively, which are calculated as follows:
  (4) $$E_{Rx}=E_{elect}\times k$$
  (5) $$E_{Tx}=E_{Rx}+\epsilon_{amp}\times k\times d^{2}$$
  where: $\epsilon_{amp}$ and $E_{elect}$ are Transmit amplifier and Electronics Energy, respectively. *k* and *d* are bit message communicated and distance between two nodes, respectively. In this paper, only transmit and received modes were utilized because they represent the main energy consuming modes in any communication process. A node with the highest remaining energy is deemed as the most reliable.

These three locally generated parameters provide external information about a node in the network, each of them can be used (independently) to make forwarding decisions in the protocol. However, in this paper, all the parameters are simultaneously utilized for selecting a forwarding node based on reliability theory. The proposed parameters are processed using the FLS to enable selection of an appropriate node.

### 4.2. Fuzzy Logic System (FLS) Design in FuGeF

FLS stems from Computational Intelligent Systems (CIS) which are emerging technologies that can be merged with most existing systems to improve performance. These systems possess adaptive mechanisms that are capable of enabling intelligent behavior in complex and unstable environments [32]. Thus, the nature of sensor node deployment in inaccessible environment together with its resource constraints (bandwidth, energy, storage) necessitates the need for a lasting collaboration with the CIS. FLS is among the numerous CIS known for its minimal demand of memory and processing power, thereby making it a valuable mechanism for resource bound security solution. Its flexibility to accommodate natural language and logical reasoning (which are of human nature) to match any set of input-output data with minimum processing intelligence, makes it preferable in sensor-based systems since it requires minimum control [33].

**Figure 3:**
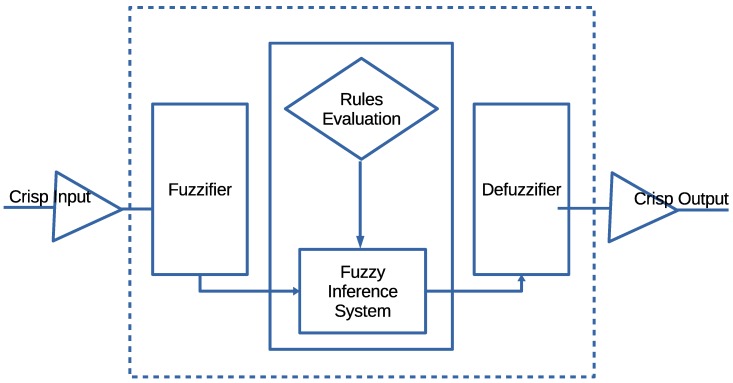
Fuzzy Logic System (FLS)

In this paper, the objective of our fuzzy logic routine is to determine the chance value for each node that responds with CTS. The FLS is composed of four main components as shown in Figure 3. We now detail the routine operation of the components.

**Figure 4:**
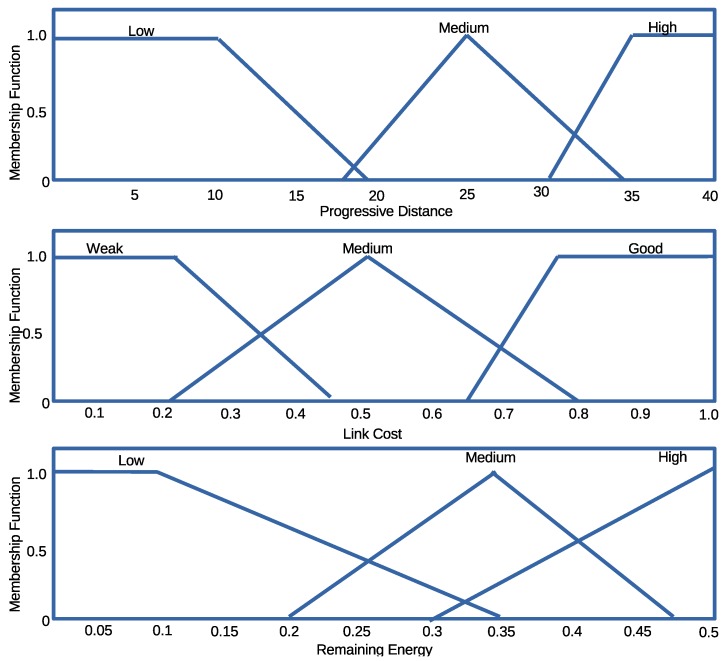
Fuzzy input membership mapping

- *The Fuzzifier:* The crisp input values from each responding node (Progressive distance, Remaining Energy, and Link cost) are passed to the Fuzzifier as shown in Figure 3. The Fuzzifier converts the input values into suitable linguistic values by mapping each value into corresponding universal set as shown in Figure 4. For instance, if *X* is the universal set, its elements are denoted by *x* such that the fuzzy set *A* in *X* is a set
  (6) $$A=x,\mu_{A}\left(x\right)|x\in X$$
  where $\mu_{A}\left(x\right)$ is a membership function (MF) of *x* in A, the function maps each element of *X* to a value between 0 and 1. The value of the membership function is the intersection point of the value of our parameters with the degree of the membership function [34].
- *Fuzzy Inference System (FIS) and Rule Evaluation:* FIS and rule evaluation components work together. FIS interprets the membership values by applying the IF-THEN rules responsible for network dynamics to obtain the fuzzy output set [35]. The commonly used Mamdani fuzzy inference technique [36] is adopted due to its simplicity to accommodate the 3-input (parameters), to produce 1-output, in the case of each CTS response. All outputs evaluated using the twenty seven (27) IF-THEN rules are gathered and combined to form a new output set.
- *Defuzzifier:* The Defuzzifier is responsible for converting the fuzzy set outputs back to crisp output, using a process known as Defuzzification. The process employs a centroid based defuzzification technique to map the output set space into the crisp output. The centroid otherwise known as the Center of Area (CoA) uses sub-divided area’s center and assigns a particular value based on the overall contribution to infer the CoA [37]. The crisp output is mathematically computed from Equation (7)
  (7) $$Output=\frac{\int \mu (x)\times xdx}{\int \mu (x)dx}$$
  where μ(x) is the aggregated membership function of the fuzzy set and *x* is the output variable. The node with the highest output or chance value is presumed to be the most appropriate node among the received replies.

### 4.3. Protocol Analysis

**Theorem 1.**  The Overhead complexity of the control messages in the network is O(N).

**Proof.**  To determine the overhead complexity in FuGeF, suppose at the beginning of each forwarding process, there are N-1 nodes within the range of the sending node, such that N-K $ORTS_{msg}$ is broadcasted to these nodes. The candidate nodes, in response to the received message, each sends C-K $CTS_{msg}$ as well as a *C* $ACK_{msg}$ from the selected candidate to the sending node. Thus the total number of control message in the network is (N-K)+(C-K)+C=N-2K+2C. Therefore, the overhead complexity of the control messages in the network is O(N) ☐

**Theorem 2.**  The Space complexity for selection process in the network is O(1)

**Proof.**  FuGeF returns a single value for each node after processing the returned response for node selection. The returned chance value for each node is compared and possibly substituted until the best chance value is determined and chosen as the next hop. Therefore, the space complexity of the selection process in the network is O(1). ☐

**Theorem 3.**  Unpredictability and response collection is ensured

**Proof.**  A fixed length collection window, if long enough, gives predictability and ensures response collection, while a dynamic (random) length collection window ensures unpredictability with no guarantee for response collection. FuGeF utilizes a pseudorandom based dynamic collection window. which exhibits dynamism within a certain fixed interval to ensure unpredictability and response collection. ☐

## 5. Performance Evaluation

This section evaluates the performance of the DWSIGF and the proposed FuGeF protocol using MATLAB simulation. The protocols were evaluated in terms of packet delivery ratio, end-to-end delay, energy consumption and probability of choosing an attacker metrics. The simulation parameters used were radio bandwidth, payload size and Constant Bit Rate (CBR) streams as 200 kbps, 32 bytes, and 100 packets respectively, to emulate the type of traffic expected in low-bandwidth networks. The simulation setting presented in Table 1 was used for all experiments and assumptions made in [3,38] were maintained. In the simulation, a terrain of 150 square meters containing 196 sensor nodes, each having a communication range of 40 m, was prepared and the Gaussian distribution with standard deviation of four meters was used over the terrain to achieve uniform node distribution for the network.

### 5.1. Attack-Based Network.

#### 5.1.1. Attack Model and Assumptions

We chose to utilize the Blackhole with CTS rushing attack model because it is considered a spatio-temporal form of attack due to the following features:

- The attackers can analyze a network’s packet propagation behavior, thereby obtaining information regarding vicinity and position of other nodes in the network.
- The attackers can also monitor the timing semantics of a routing protocol. This capability enables them to know when a node is about to communicate.

These two distinct capabilities enable the attacker to perform an effective intersection attack with no regard for the protocol timing semantics, especially when CTS responses are being sent to the sending node during the collection window period. Thus, presenting itself both in position (Spatially) and time (temporally) as the most suitable forwarding candidate, which if selected, drops all received packets and eventually halts the routing process. For the experiments ahead, we targeted a static network where we tested the impact of attackers using two scenarios:

1. Strategic Placement of Attackers.
2. Random Placement of Increasing Attackers.

#### 5.1.2. Strategic Placement of Attacker

**Figure 5:**
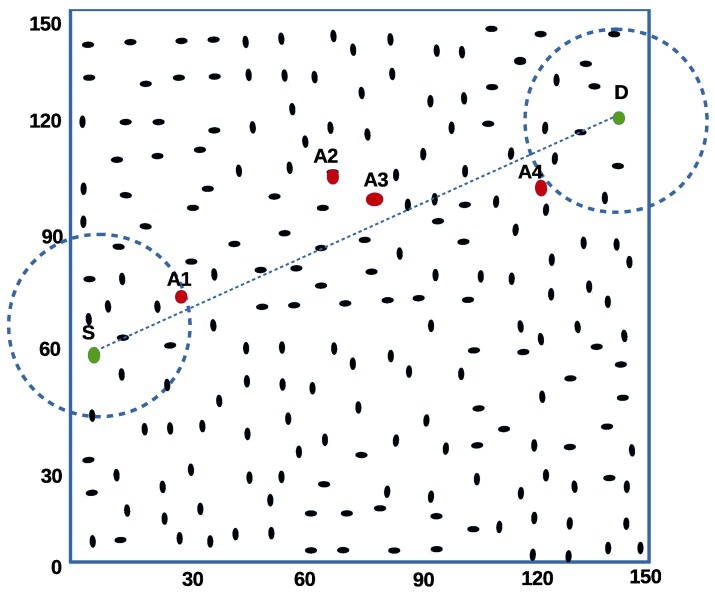
Strategic placement of malicious nodes

In this experiment, malicious nodes are placed along the path to the destination as seen in Figure 5. Point A1 and A4 are known as optimal positions. They are known to be points with the highest correlation to the first and last hop respectively. A2 and A3 are considered modal positions because they present average correlation of being selected as middle hop nodes existing between the first and last hops.

**Figure 6:**
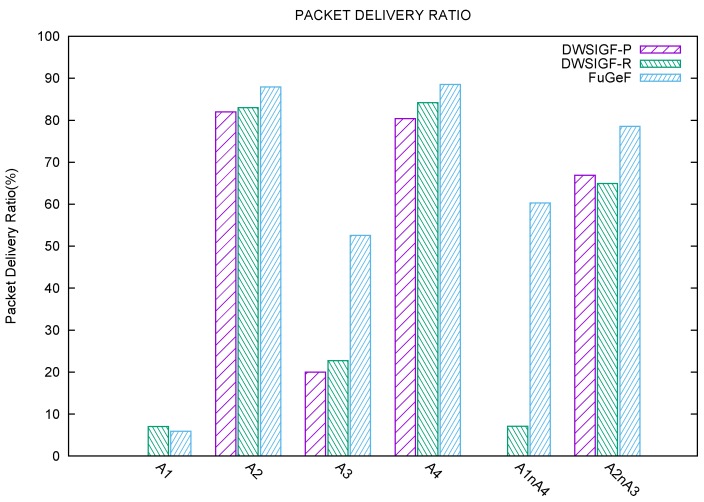
Packet delivery.

**Figure 7:**
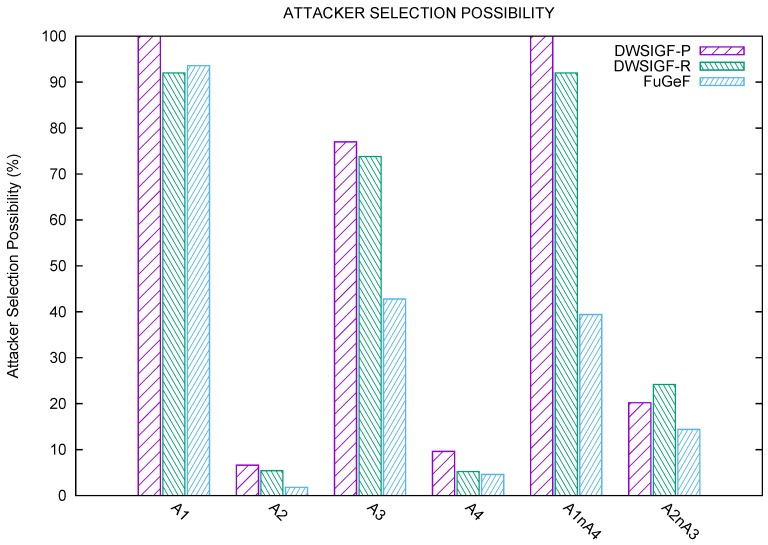
Possibility of attacker selection.

The experiment is set up to test one-to-one single CBR stream to eliminate the impact of network congestion. The direct line between the sending node S (left) and destination D (right) shown in Figure 5. is drawn to show the optimal and modal strategic node positions as they lie across the route to node D. The experiment measures six sub-scenario to determine the effect of attackers and their locations on packet delivery ratio. Results shown in the graphs Figure 6 and Figure 7 are the average of 500 simulation runs for each attacker/attackers position.

#### 5.1.3. Single Attacker

- *Attacker A1:* It can be observed from Figure 6 and Figure 7 that this optimally positioned attacker poses a serious threat to all protocols. DWSIGF-P has zero chance of bypassing such a treat due to its predictable forwarding strategy. DWSIGF- R with 7% slightly outperforms FuGeF with 6% packet delivery. This is because the FLS consensus bearly notices changes in initial value parameters yielded among all nodes. These poor performances were observed in all protocols due to the high correlation the maliciously placed node has to the first hop.
- *Attacker A2:* The modal positioned attacker A2, unlike A1, has a small impact on the forwarding node selection. This is because the position is easily bypassed when the first hop chooses the highest distance towards the destination. DWSIGF-P, DWSIGF-R, and FuGeF were able to achieve 82%, 83% and 88% packet delivery, respectively, as shown in Figure 6. FuGeF, with 2% possibility of choosing the attacker (Figure 7), outperforms both DWSIGF-P and DWSIG-R that have 7% and 5% respectively.
- *Attacker A3*: This modal attacker shows a significant impact on the network performance. A3 has a higher chance of being selected than A2 during the routing process. 77%, 74% and 43% possibility of choosing the attacker was observed, leading to 20%, 23% and 53% packet delivery in DWSIGF-P, DWSIGF-R, and FuGeF, respectively, as shown in Figure 6 and Figure 7.
- *Attacker A4:* The attacker A4 is presumed to be the last hop node before the final destination. Due to network density, the impact of this optimal attacker was minimal such that DWSIGF-P, DWSIGF-R, and FuGeF achieved 80%, 84%, and 88% packet delivery (Figure 6) with 10%, 5% and 4.5% possibility of choosing the attacker (Figure 7), respectively.

#### 5.1.4. Multiple Attackers

- *Attacker A1 and A4:* further experiments were conducted by simultaneously subjecting the network to the optimally positioned attackers, A1 and A4. From Figure 6, it is shown that DWSIGF-P was barred completely from routing due to its selection strategy as A1 was chosen in all attempts. DWSIGF-R achieved 7% packet delivery with a 92% chance of selecting the attacker. FuGeF, however, succeeded in achieving 60% packet delivery with 40% chance of selecting the attacker.
- *Attacker A2 and A3:* Both malicious nodes in the modal positions were simultaneously tested. DWSIGF-P, DWSIGF-R, and FuGeF were able to achieve 67%, 65% and 79% packet delivery with 20%, 24% and 14% possibility in choosing an attacker respectively, as shown in Figure 6 and Figure 7.

#### 5.1.5. Random Placement of Increasing Attackers

**Figure 8:**
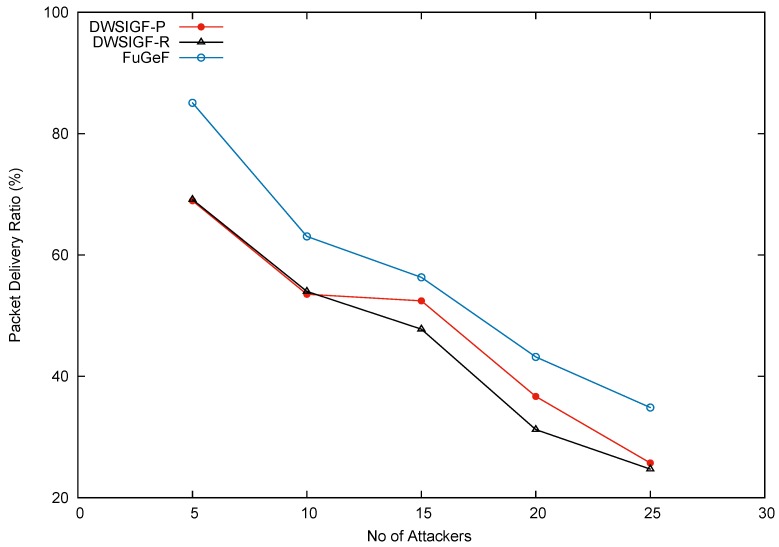
Packet delivery.

In this experiment, multiple senders (sources) and receivers (destinations) were tested in the presence of increasing multiple attackers within the network. The set up comprised of 6 random nodes from the left side of the terrain, representing the locations of events of interest. The nodes were tasked with sending 6 CBR flows to 2 random nodes on the right-hand side of the terrain representing the destinations (sink nodes), such that each node receives 3 CBR flows from the 6 sending nodes. Results shown in the graph (Figure 8) are the average of 100 simulation runs for each increasing number of attackers.

Figure 8 shows successful packet delivery plotted against an increasing number of attackers randomly deployed in the network. DWSIGF-P employs greedy forwarding strategy that could easily bypass attackers as long as they do not fall on the direct line centered at the sextant towards the destination. This directed forwarding enabled DWSIGF-P to show better performance than DWSIGF-R. DWSIGF-R on the other hand uses random forwarding; this random propagation influence the frequent selection of randomly placed attackers, causing substantial packet losses during the routing process. The unrivaled performance in FuGeF is attributed to its spontaneous and yet directed propagation pattern. The pattern was as a result of FLS consensus after processing nodes parameters (Progressive distance, connectivity cost, and remaining energy) as they change during the routing process. FuGeF forwarding strategy emerged to be more reliable than both DWSIGF-P and DWSIGF-R (they only utilize the predictive IDTD parameter for forwarding).

#### 5.1.6. Experimental Conclusions on Attack-Based Network

FuGeF’s performance in minimizing the possibility of choosing an attacker in scenarios explained in Section 5.1.2 and Section 5.1.5, was mainly due to the dynamism employed and its spontaneous selection scheme. The dynamism (pseudo-random) adds a slight shift to the normal routing semantics. This eventually causes unpredictability in timing and selection of the forwarding node, thus abating attacker’s effect. In DWSIGF, a random time(ms) is employed for the collection window. Rushing a CTS signal by the attacker is often presumed to be successful as the timing lacks fixed interval for collection to begin. In the case of FuGeF, the pseudo-random time utilized to collect CTS reply has a given time interval. This interval causes a slight delay in the start of a collection which prevents the CTS rushing attacker from participating in the selection process due to its early arrival.

Additionally, spontaneity in FuGeF’s selection scheme caused by FLS consensus (based on node’s remaining energy, connectivity cost, and progressive distance) has also influenced the protocol’s resistivity to attacks. This is because if a drop is noticed in any of the parameters (associated to a node), FuGeF disregards such a node until all its neighboring nodes are of equal or lesser value than the selected node. Thus, the drop noticed in malicious node’s parameters that have been repeatedly selected would influence the selection of other non-malicious nodes during the routing process.

Finally, FuGeF’s packet delivery averagely outperforms DWSIGF-P and DWSIGF-R by 48.80% and 25.55%, and, 16.44% and 21.00% for strategic (Section 5.1.2) and random (Section 5.1.5) based attack scenario, respectively.

### 5.2. Performance in Attack-Free Network (Base Performance)

In this scenario, we test the many-to-many CBR flows to show protocol performance in an attack-free network to illustrate the base performance. In the experiments, 6 nodes from the left side of the terrain representing the locations of events of interest were randomly selected, The nodes were tasked with sending 6 CBR flows to 2 nodes on the right hand side of the terrain representing the base station such that each node receives 3 CBR flows from the sending nodes. Results shown in the graphs are the average of 100 simulation runs for each traffic load/CBR flow.

**Figure 9:**
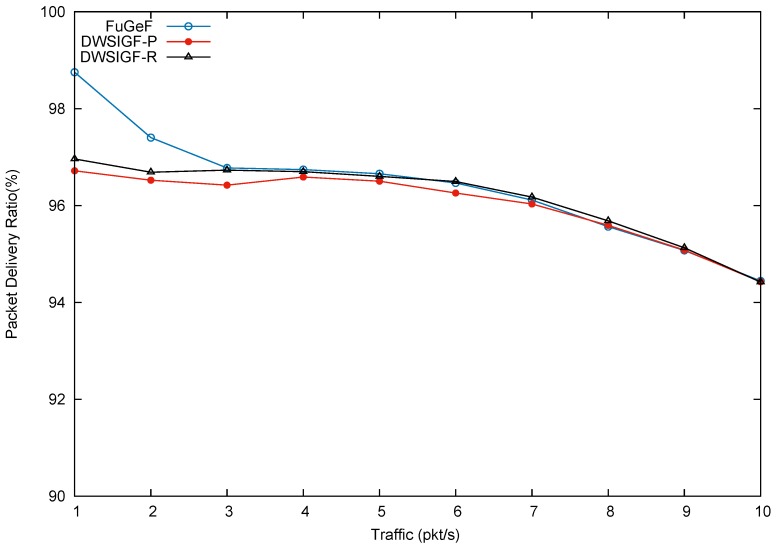
Packet Delivery Ratio (PDR).

**Figure 10:**
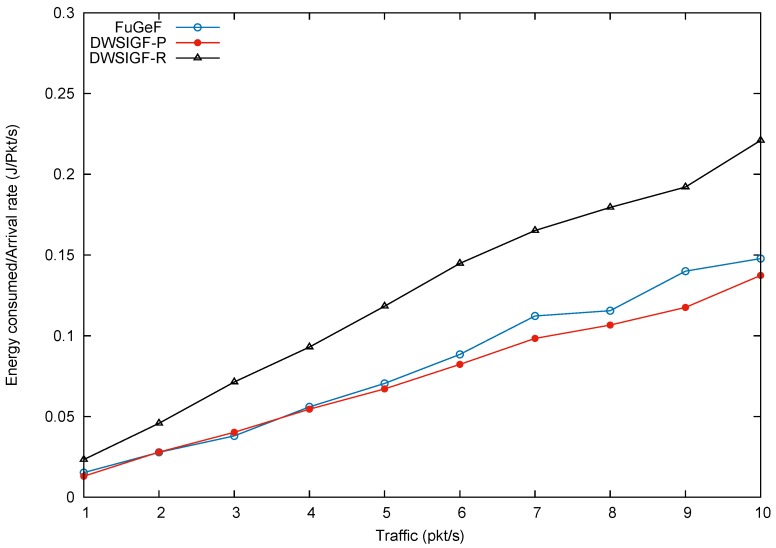
Energy consumption.

**Figure 11:**
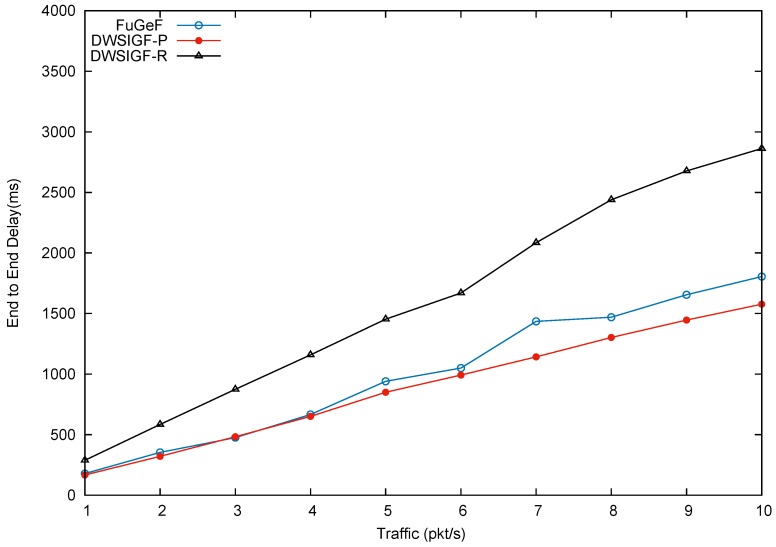
End to end delay.

Figure 9, Figure 10 and Figure 11, show packet delivery ratio, energy consumption and end-to-end delay for DWSIGF-P, DWSIGF-R, and the proposed FuGeF. Figure 9 illustrates that the proposed FuGeF at lower traffic (1–4 pkt/s) achieved a higher PDR, while maintaining lower energy consumption and end-to-end delay as shown in Figure 10 and Figure 11. This was due to the ability of the FLS to determine nodes with good enough links (among the forwarding candidates), that have made significant progress towards the destination. The good links ensured that substantial packet loss was mitigated and the significant progress enabled utilization of few hops to reach the destination, resulting in reduced energy consumption and delay. As the traffic increased from 5–10 pkt/s, congestion and interference also increased. FuGeF tries to maintain optimal performance by resorting to choosing optimal nodes that are less prone to interference in order to mitigate packet loss. This, however, increased the number of hops needed to reach the destination, which in turn causes the increase in energy consumption and end-to-end delay as shown in Figure 10 and Figure 11, respectively.

DWSIGF-P, unlike FuGeF, employs the greedy forwarding which selects nodes that made the highest progress towards the destination, disregarding their connectivity cost. Although the DWSIGF-P is vulnerable to interference, its selection utilizes few hops which maintained lower energy consumption and end-to-end delay as shown in Figure 10 and Figure 11, respectively. However, it endures substantial packet loss all through from lower to higher traffic as illustrated in Figure 9.

DWSIGF-R chooses nodes randomly from the forwarding area. The random selection may result to significant progress or lesser progress made towards the destination. Lesser progress incurs increased number of hops, which causes overlap of vicinities (range–distance transversed overlap). Nodes which fall between this overlap are exposed to multiple overhear of transmissions. This increases the amount of energy consumed and end-to-end delay as shown in Figure 10 and Figure 11, respectively. However, it maintained a higher packet delivery when compared to DWSIGF-P, due to shorter distance utilized which are less vulnerable to interference.

Overall, it can be inferred that despite the average performance displayed by FuGeF in an attack-free network, its trade-off in security and normal routing operation (base performance) still outperforms DWSIGF-P and DWSIGF-R according to the concept of the resource bound security solution. The concept, which states that pertinent mechanism installed in the protocols that are kept active to provide security, should not degrade the base performance to an unacceptable level. On this note, it can be observed that DWSIGF-P base performance was able to utilize minimum energy and time(end to end delay) required for routing. However, it endured substantial packet losses and provided minimal security that was inadequate. DWSIGF-R security performance and packet delivery (base performance) were achieved at a heightened cost of energy and time. FuGeF made optimal utilization of energy and time for its normal routing operation. It further provided an unrivaled performance in security which prevented substantial packet losses to the network. This has made FuGeF the most suitable candidate protocol for an effective resource bound security solution for WSN.

## 6. Conclusions

In an effort to mitigate the effect of spatio-temporal attacks on a network, resource bound security solution suggested altering protocol semantics or installing mechanisms that would prevent the selection of malicious entity as well as maintain base performance of the protocol. In this paper, a Fuzzy-based Geographic Forwarding (FuGeF) protocol has been presented for WSN. It utilizes a pseudo-random form of dynamism and relies on parameters which include: progressive distance, connectivity cost, and remaining energy as well as a fuzzy logic system for node selection. Extensive simulation experiments were conducted to evaluate the effectiveness of the proposed FuGeF. The results show that FuGeF in an attack based network significantly outperforms DWSIGF-P and DWSIGF-R in terms of packet delivery. It also achieves a better trade-off between security and base performance. Future works lie in strengthening FuGeF in an attempt to thwart more devastating security threats to a network, and providing comprehensive results (theoretical and practical).

## Figures and Tables

**Table 1:** Simulation parameters.

System Parameter	Value
Terrain	150 × 150 m
Number of Nodes	196, Uniform
Radio Range	40 m
Application Streams	CBR
Radio Bandwidth	200 kbps
Simulation Length	100 packets
Node Placement	Grid +N(0,16) noise
